# ﻿The Oriental millipede genus *Nepalella* Shear, 1979, with the description of a new species from Thailand and an updated key (Diplopoda, Chordeumatida, Megalotylidae)

**DOI:** 10.3897/zookeys.1084.78744

**Published:** 2022-02-01

**Authors:** Natdanai Likhitrakarn, Sergei I. Golovatch, Somsak Panha

**Affiliations:** 1 Division of Plant Protection, Faculty of Agricultural Production, Maejo University, Chiang Mai, 50290, Thailand; 2 Biodiversity and Utilization Research Center of Maejo University, Maejo University, Chiang Mai, 50290, Thailand; 3 Institute for Problems of Ecology and Evolution, Russian Academy of Sciences, Leninsky pr. 33, Moscow 119071, Russia; 4 Animal Systematics Research Unit, Department of Biology, Faculty of Science, Chulalongkorn University, Bangkok, 10330, Thailand; 5 Academy of Science, The Royal Society of Thailand, Bangkok 10300, Thailand

**Keywords:** Distribution, Indochina, key, taxonomy

## Abstract

The Oriental genus *Nepalella* is reviewed, rediagnosed and shown to comprise 28 species, including *N.siamensis***sp. nov.** from southeastern Thailand. All *Nepalella* species are keyed, and their distributions mapped, being highly localized and mainly allopatric. Unlike most congeners, which are largely confined to subtropical environments (including montane to high-montane conditions, up to 3800 m a.s.l.) or karst caves (eight species, all in southern China alone), the new species is the southernmost in the distribution area of the entire genus, also being among the very few (four) that are restricted to lowland, purely tropical habitats.

## ﻿Introduction

*Nepalella* Shear, 1979 is one of the relatively few Indo-Malayan genera of the millipede order Chordeunatida and only the second in the small family Megalotylidae (Enghoff et al. 2015). Unlike the oligotypic, more boreal, East Asian *Megalotyla* Golovatch, in Golovatch and Mikhaljova 1978, represented by only two species from the Russian Far East or North Korea, *Nepalella* is far more southerly in distribution, being also regarded as one of the most species-rich diplopod genera in the entire Oriental Realm (Golovatch et al. 2006b).

**Figure 1.**
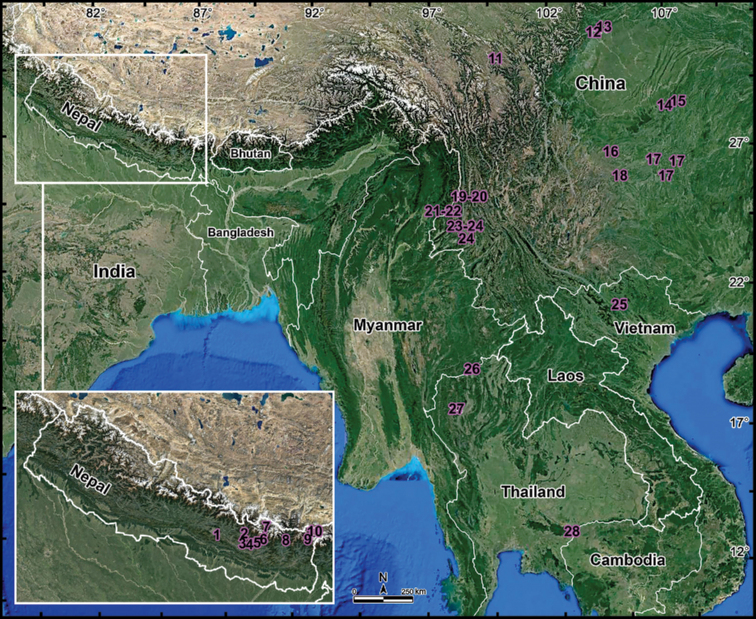
Distributions of *Nepalella* species (28 species), arranged from northwest to southeast **1***N.phulcokia* Mauriès, 1988 **2***N.gairiensis* Mauriès, 1988 **3***N.thodunga* Shear, 1979 **4***N.deharvengi* Mauriès, 1988 **5***N.ringmoensis* Mauriès, 1988 **6***N.tragsindola* Mauriès, 1988 **7***N.khumbua* Shear, 1979 **8***N.jaljalae* Mauriès, 1988 **9***N.taplejunga* Shear, 1987 **10***N.gunsa* Shear, 1987 **11***N.marmorata* Golovatch, Geoffroy & Mauriès, 2006 **12***N.grandoides* Golovatch, Geoffroy & Mauriès, 2006 **13***N.lobata* Liu, in Liu et al. 2017 **14***N.jinfoshan* Liu, in Liu et al. 2017 **15***N.wangi* Liu, in Liu et al. 2017 **16***N.grandis* Golovatch, Geoffroy & Mauriès, 2006 **17***N.troglodytes* Liu, in Liu et al. 2017 **18***N.caeca* Shear, 1999 **19***N.kavanaughi* Shear, 2002 **20***N.pianma* Shear, 2002 **21***N.pallida* Mauriès, 1988 **22***N.birmanica* Mauriès, 1988 **23***N.magna* Shear, 2002 **24***N.griswoldi* Shear, 2002 **25***N.vietnamica* Golovatch, 1983 **26***N.taiensis* Mauriès, 1988 **27***N.inthanonae* Mauriès, 1988 **28***N.siamensis* sp. nov.

*Nepalella* is presently known to comprise 27 described species ranging from Nepal (10 species) in the west, southern China (12 species) in the north, through Myanmar and northern Thailand in the south (2 species each), to northern Vietnam (1 species) in the east (Liu et al. 2017b; Fig. 1). Most species of *Nepalella* are only known from a single locality, being highly localized in distribution (Table 1, Fig. 1). This concerns not only the rather numerous cavernicoles (eight species, largely presumed troglobionts confined to karst caves in southern China), but also epigean congeners, among which most are montane (>800 m a.s.l.) to high-montane (2200–3800 m a.s.l.) and allopatric (Table 1), with only two pairs that have been found to occur syntopically (Shear 2002; Liu et al. 2017b). Some *Nepalella* species are among the largest Chordeumatida globally and they mainly appear to be restricted to subtropical rather than purely tropical environments, all lying between 23.5° and 34°N (Fig. 1), whereas lowland, typically tropical encounters are only very few.

**Table 1.** Checklist of all described *Nepalella* species, arranged in alphabetic order and supplied with geographic details (Shear 1979, 1987, 1999, 2002; Golovatch 1983; Mauriès 1988; Golovatch et al. 2006a, 2006b; Liu et al. 2017b).

No.	Species	Locality
1	*Nepalellabirmanica* Mauriès, 1988	Myanmar, Kambaiti (2270 m)
2	*Nepalellacaeca* Shear, 1999	China, Guizhou Province, Shuicheng County, Cave Anjia Yan; same County, Cave Shendongmigong (26°35'15"N, 104°59'47"E, 1900 m)
3	*Nepalelladeharvengi* Mauriès, 1988	Nepal, Sagarmatha Province, trace of the Tomba-Kosi in Namche Bazar: Sété (2900–3250 m); same locality (2900 m); same locality, above Sété (3000–3300 m); same locality, Sété pass (Abies) (3000–3400 m); same locality (3300–3500 m)
4	*Nepalellagairiensis* Mauriès, 1988	Nepal, Sagarmatha Province, trace of the Tomba-Kosi in Namche Bazar: Gairi; same locality, chasse à vue
5	*Nepalellagrandis* Golovatch, Geoffroy & Mauriès, 2006a	China, Yunnan Province, Zheng Xiong County, Cave Bai Yin Dong
6	*Nepalellagrandoides* Golovatch, Geoffroy & Mauriès, 2006b	China, Sichuan Province, Beichuan County, Cave Yuan Dong; same County Cave Black Wind Dong
7	*Nepalellagriswoldi* Shear, 2002	China, Yunnan Province, Baoshan Prefecture, Mountain Gaoligong, Luoshuidong, 28 air km East of Teng Chong, (24°57'N, 98°45'E, 2300 m); same Prefecture, Mountain Gaoligong, Namkang, 36 air km Southeast of Teng Chong (24°50'N, 98°47'E, 2100 m)
8	*Nepalellagunsa* Shear, 1987	Nepal, Taplejung District, south of Gunsa (=Ghunsa), (3800–3600 m)
9	*Nepalellainthanonae* Mauriès, 1988	Thailand, Chiang Mai Province, Doi Inthanon National Park (2000–2540 m)
10	*Nepalellajaljalae* Mauriès, 1988	Nepal, Kosi Province, Jaljale Himal, forest in south of Mangsingma, 2200 m (Mauriès 1988)
11	*Nepalellajinfoshan* Liu, in Liu et al. 2017b	China, Chongqin Province, Jinfoshan, Cave Houshan Dong (28°58'44"N, 107°11'20"E, 1500 m); same locality, Cave Lingguan Dong (29°01'10"N, 107°10'28"E, 2100 m)
12	*Nepalellakavanaughi* Shear, 2002	China, Yunnan Province, Nujiang Prefecture, Pianma, Mountain Gaoligong, native forest (25°59'N, 98°40'E, 2500 m)
13	*Nepalellakhumbua* Shear, 1979	Nepal, Kumbu, Mt. Everest region, confluence of Phunki and Imja Drangka, northeast of Kumjung (3250–3300 m)
14	*Nepalellalobata* Liu, in Liu et al. 2017b	China, Sichuan Province, Mianyang City, Beichuan County, Cave Liangshui Dong (31°55'30"N, 104°40'56"E, 1000 m)
15	*Nepalellamagna* Shear, 2002	China, Yunnan Province, Baoshan Prefecture, Mountain Gaoligong, Luoshuidong, 28 air km East of Teng Chong (24°57'N, 98°45'E, 2300 m)
16	*Nepalellamarmorata* Golovatch, Geoffroy & Mauriès, 2006a	China, Sichuan Province, Zin Long County, Snake Mouth Cave; same County, Cave Three Eyes (Trois Yeux) (AKL)
17	*Nepalellapallida* Mauriès, 1988	Myanmar, Kambaiti (2270 m)
18	*Nepalellaphulcokia* Mauriès, 1988	Nepal, Kathmandu District, Phulcoki (2250 m); same locality (2650 m)
19	*Nepalellapianma* Shear, 2002	China, Yunnan Province, Nujiang Prefecture, Pianma, Mountain Gaoligong, native forest (25°59'N, 98°40'E, 2500 m)
20	*Nepalellaringmoensis* Mauriès, 1988	Nepal, Sagarmatha Province, trace of the Tomba-Kosi in Namche Bazar: Gonda (before Ringmo) (2750–3000 m)
21	*Nepalellasiamensis* sp. nov.	Thailand, Sa Kaeo Province, Ta Phraya District, Ta Phraya National Park (14°08'22"N, 102°40'11"E, 183 m)
22	*Nepalellataiensis* Mauriès, 1988	Thailand, Chiang Mai Province, Doi Pha Hom Pok, northwest of Fang (1550–1750 m)
23	*Nepalellataplejunga* Shear, 1987	Nepal, Taplejung District, ridge Lasse Dhara and pasture Lassetham (3000–3300 m)
24	*Nepalellathodunga* Shear, 1979	Nepal, Thodung near Jiri and Those (3200 m)
25	*Nepalellatragsindola* Mauriès, 1988	Nepal, Sagarmatha Province, trace of the Tomba-Kosi in Namche Bazar: east of Tragsindo-La (2450–2650 m)
26	*Nepalellatroglodytes* Liu, in Liu et al. 2017b	China, Guizhou Province, Guiyang City, Xifeng County, Hejiadong Village, Cave Hejia Dong (27°02'31"N, 106°31'40"E, 1200 m); same county, Mushan Village, Cave Zhangkou Dong (27°04'10"N, 106°32'55"E, 1300 m); same province, Qiannan Zizhizhou, Longli County, Cave Feilong Dong (26°27'11"N, 106°58'46"E, 1200 m); same province, Qiannan Zizhizhou, Fuquan County, Cave Sanlou Dong (26°56'46"N, 107°18'47"E, 1280 m)
27	*Nepalellavietnamica* Golovatch, 1983	Vietnam, Yen Bai Province, Chay valley, Lục Yên (300 m)
28	*Nepalellawangi* Liu, in Liu et al. 2017b	China, Chongqin Province, Wulong County, Huangying Town, Qimenxia, Cave I Dong (29°10'33"N, 107°42'12"E, 1300 m)

Therefore, the discovery of another lowland, tropical species of *Nepalella*, this time in southeastern Thailand, is noteworthy, especially as it represents both the southernmost and the most lowland congener reported to date. The new species was collected in a dipterocarp forest in the Ta Phraya National Park, Sa Kaeo Province, Thailand (Fig. 1). The opportunity is also taken to update the previous key to *Nepalella* spp. (Golovatch et al. 2006b) and to revisit its taxonomy and distribution.

## ﻿Materials and methods

Material was euthanized using a two-step method following Guidelines for the Euthanasia of Animals (AVMA 2013). Specimens were then preserved in 75% ethanol for morphological observations which were carried out in the laboratory. The specimens were examined, measured and photographed under a Nikon SMZ 745T trinocular stereo microscope, equipped with a Canon EOS 5DS R digital SLR camera. Digital images obtained were processed and edited with Adobe Photoshop CS5. Line drawings were based on photographs and examined under a stereo microscope equipped with a digital SLR camera. Scanning electron micrographs (**SEM**) of gonopods coated with a 8 nm gold layer using a CCU-010 high vacuum sputter and a carbon coater (Safematic) were imaged with a TESCAN VEGA3 scanning electron microscope operated at 5 keV of acceleration voltage and returned to alcohol after SEM examination. The images were enhanced and arranged in plates with Adobe Photoshop CS6 software. Collecting sites were located by GPS WGS84 datum using a Garmin GPSMAP 60 CSx, and all coordinates and elevations were checked with Google Earth. The holotype of *Nepalellasiamensis* sp. nov. is housed in the Museum of Zoology, Chulalongkorn University (**CUMZ**), Bangkok, Thailand. The Animal Care and Use Protocol Review No. 1723018 was applied.

In the synonymy sections, D stands for the original description and/or subsequent descriptive notes, K for the appearance in a key, L for the appearance in a species list, and M for a mention.

Terminology concerning gonopodal and somatic structures, including the following abbreviations used in the text, mostly follows Spelda (2001), Golovatch et al. (2006a) and Liu et al. (2017b).

Abbreviations of certain gonopodal structures in the figures are explained both in the text and figure captions.

**CIX** macrochaetal index; distance between the exterior and median macrochaeta divided by the distance between the interior and median macrocheata;

**MA** macrochaetal angle; formed between the arm from the median and exterior macrochaetae and that between the median and interior macrochaetae;

**MIX** median index; distance between the interior macrochaeta and axial (longitudinal) suture divided by the distance between the interior and median macrochaeta;

**PIX** paraterga index; distance between the edges of both pataterga and the edges of the prozonite divided by double the length of a paratergum.

## ﻿Taxonomy

### ﻿Family Megalotylidae Golovatch, in Golovatch and Mikhaljova 1978

#### ﻿Genus Nepalella

Taxon classificationAnimaliaChordeumatidaMegalotylidae

Shear, 1979

F6C451BF-0C8C-50FF-B65B-8C2E5463D1B5

Nepalella Shear, 1979: 126, D, K.
Nepalella – Golovatch 1983: 126, D; Shear 1987: 237, D; 1999: 2, D; 2002: 65, D; Mauriès 1988: 26, D; Golovatch et al. 2006a: 83, M, K; 2006b: 84, M; Liu et al. 2017b: 455, M, K; Golovatch and Liu 2020, L, M.

##### Diagnosis.

The millipede genus *Nepalella* Shear, 1979 as a member of the family Megalotylidae is mainly distinguished from *Megalotyla*, the only other component genus of the family, by the anterior gonopods still showing weakly developed coxites placed on a relatively small, central sternum (versus coxites completely absent from a larger sternal plate in *Megalotyla*) (Enghoff et al. 2015).

##### Brief description.

Body medium- to large-sized (ca 10–42 mm long, ca 0.64–3.2 mm wide), with 28 or 30 segments. Mentum not divided. Paraterga either distinct keels or small bulges, or missing. ♂ legs 3–7 often distinctly and increasingly crassate, some with femoral knobs. ♂ legs 10 with coxal glands, but ♂ legs 11 either with or without coxal glands. Female genitalia often species-characteristic.

Anterior gonopods strongly reduced, consisting of only a small sternal (coxosternal?) plate with a median lamellate process and two lateral spikes (coxites). Posterior gonopods with large and bipartite coxites, divisions being clearly visible when seen in anterior view, either branching or simple; lateral division often in the form of a broad, flat plate turned with its axis parallel to body midline. Posteriorly, at least one branch covered with fine cuticular fimbriae present, entire posterior surface of coxite may appear densely hairy. Telopodites may be quite small, typically reduced to a prefemur and a femur, the latter turned sharply dorsad.

##### Type species.

*Nepalellakhumbua* Shear, 1979, by original designation.

##### Other species included.

*Nepalellabirmanica* Mauriès, 1988, *N.caeca* Shear, 1999, *N.deharvengi* Mauriès, 1988, *N.gairiensis* Mauriès, 1988, *N.grandis* Golovatch, Geoffroy & Mauriès, 2006, *N.grandoides* Golovatch, Geoffroy & Mauriès, 2006, *N.griswoldi* Shear, 2002, *N.gunsa* Shear, 1987, *N.inthanonae* Mauriès, 1988, *N.jaljalae* Mauriès, 1988, *N.jinfoshan* Liu, in Liu et al. 2017b, *N.kavanaughi* Shear, 2002, *N.lobata* Liu in Liu et al. 2017b, *N.magna* Shear, 2002, *N.marmorata* Golovatch, Geoffroy & Mauriès, 2006, *N.pallida* Mauriès, 1988, *N.phulcokia* Mauriès, 1988, *N.pianma* Shear, 2002, *N.ringmoensis* Mauriès, 1988, *N.taiensis* Mauriès, 1988, *N.taplejunga* Shear, 1987, *N.thodunga* Shear, 1979, *N.tragsindola* Mauriès, 1988, *N.troglodytes* Liu, in Liu et al. 2017b, *N.vietnamica* Golovatch, 1983, *N.wangi* Liu, in Liu et al. 2017b, *N.siamensis* sp. nov.

##### Distribution.

Nepal, southern China, Myanmar, northern and southeastern Thailand, and northern Vietnam (Fig. 1).

##### A brief historical account.

The genus *Nepalella* was first established by Shear (1979), based on two new species from Nepal, including characters of the female vulvae (= cyphopods) added to both descriptions. Golovatch (1983) described a new species from northern Vietnam and, together with *Megalotyla*, assigned it to the family Megalotylidae. Shear (1987) added further two new species from Nepal, this time using only male specimens for descriptions.

Mauriès (1988) published ten new *Nepalella* species from Nepal, Myanmar or Thailand, including descriptions of female genitalia that followed Shear’s (1979) pattern. Although the morphological differences in the vulvae were often found species-specific, Mauriès (1988) preferred not to describe new species based solely on female material.

Shear (1999, 2002) reviewed *Nepalella* and described five new species from China, including *N.magna*, the first to be named based on four female specimens alone. That species was particularly large in size, showed morphologically distinctive vulvae, and found coexisting in syntopy with both *N.griswoldi* and *Vieteumalongi* Shear, 2002, the latter taxon another chordeumatidan genus and family (Shear 2002).

Golovatch et al. (2006a, b) described a further three *Nepalella* from Chinese caves and provided a key to all species then known in the genus. More recently, Liu et al. (2017b) published four new species and two new records of *Nepalella*, including a key to, and a distribution map for, all 12 species of *Nepalella* from China. This latter study also pioneered barcoding in *Nepalella*, providing the first molecular-based phylogeny of a chordeumatidan genus outside Europe.

### ﻿Description of a new species

#### ﻿ Nepalella siamensis sp. nov.

Taxon classificationAnimaliaChordeumatidaMegalotylidae

D613257E-4D9C-5D2A-92E2-134C97B764E6

http://zoobank.org/3768467C-2FB4-4E2F-88A8-3977AE5ADDFF

Figs 2 , 3 , 4 , 5

##### Holotype.

♂ (CUMZ), Thailand, Sa Kaeo Province, Ta Phraya District, Ta Phraya National Park, 183 m a.s.l., 14°08'22"N, 102°40'11"E, 27.10.2010, leg. N. Likhitrakarn and S.I. Golovatch. The holotype of *Nepalellasiamensis* sp. nov. is housed in the Museum of Zoology, Chulalongkorn University (CUMZ), Bangkok, Thailand.

##### Etymology.

To emphasize “Siam”, referring to the former name of Thailand as the *terra typica*; adjective.

**Figure 2.**
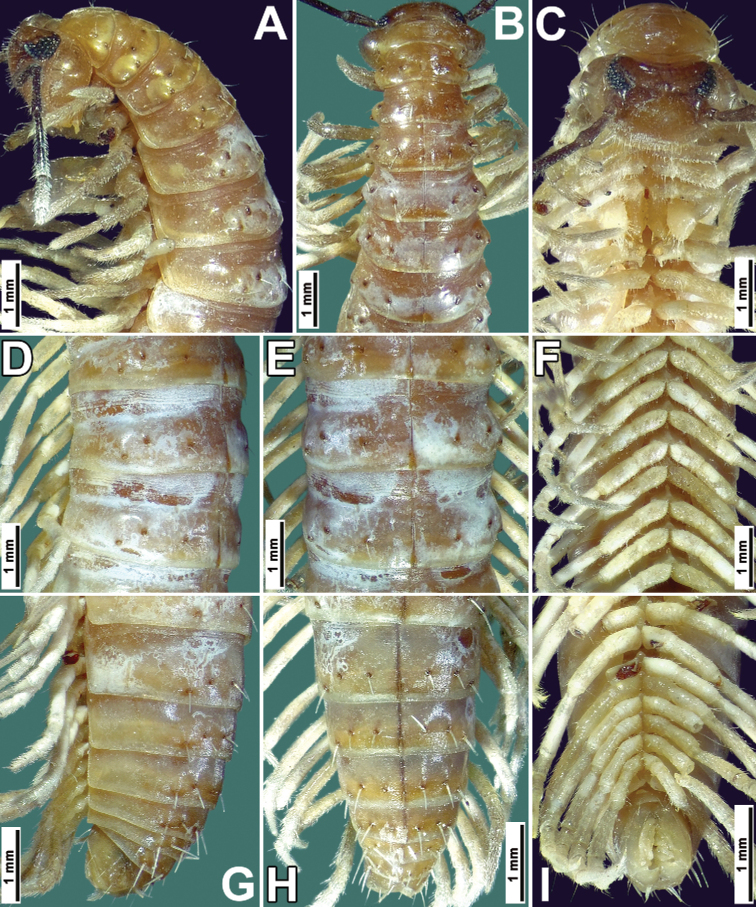
*Nepalellasiamensis* sp. nov., ♂ holotype (CUMZ) **A–C** anterior part of body, lateral, dorsal and ventral views, respectively **D–F** body segments 8–10, sublateral, dorsal and ventral views, respectively **G–I** posterior part of body, lateral, dorsal and ventral views, respectively.

##### Diagnosis.

**Figure 3.**
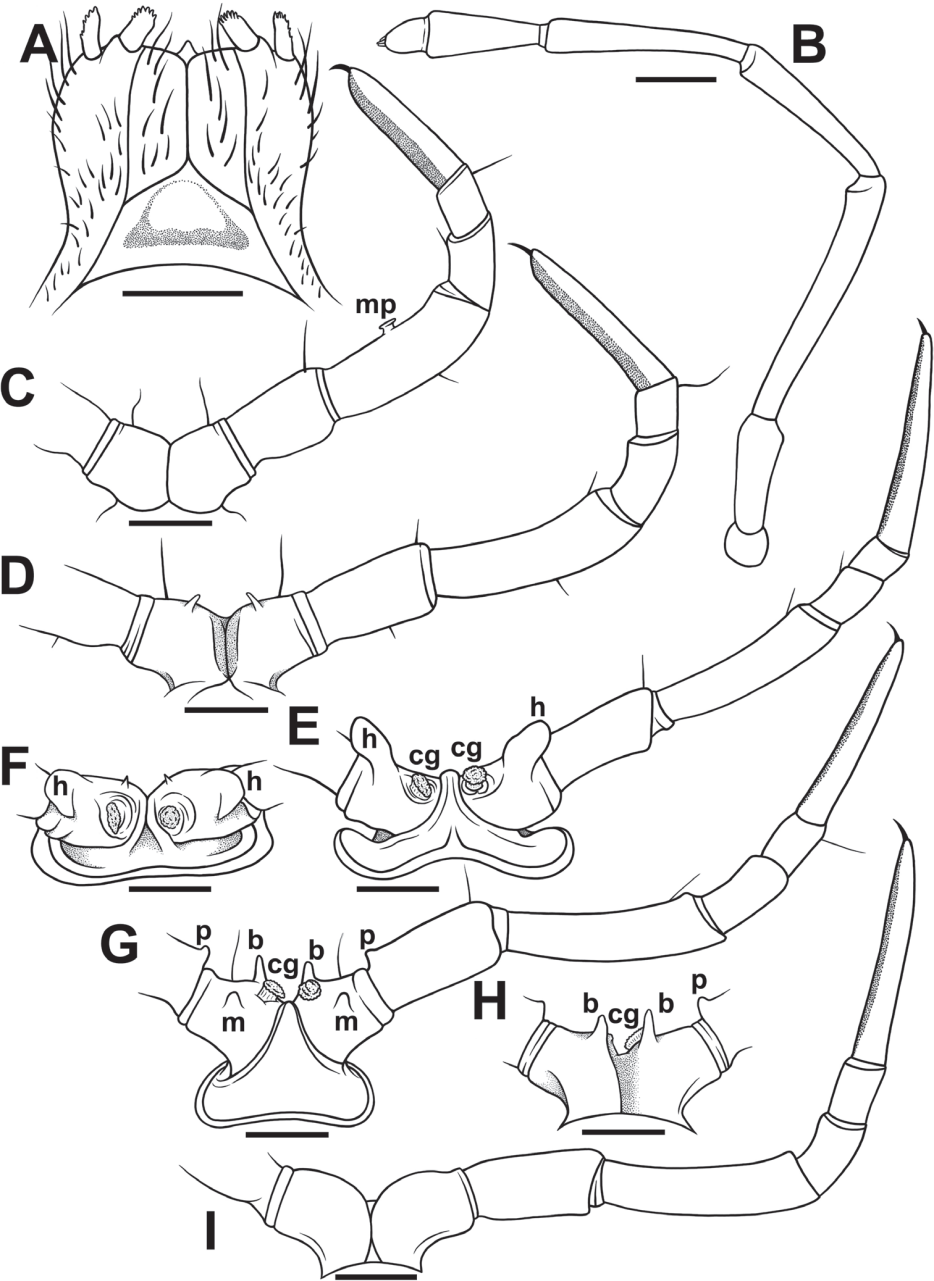
*Nepalellasiamensis* sp. nov., ♂ holotype (CUMZ) **A** gnathochilarium, ventral view **B** antenna **C** leg 4, caudal view **D** leg 7, caudal view **E** leg 10, front view **F** coxa 10, subcaudal view **G** leg 11, front view **H** coxa 11, caudal view **I** leg 12, caudal view. Abbreviations: **b** basal process, **cg** coxal gland, **m** medial process, **mp** mushroom-shaped protuberance, **p** parabasal process, **h** horn-shaped process. Scale bars: 0.25 mm.

**Figure 4.**
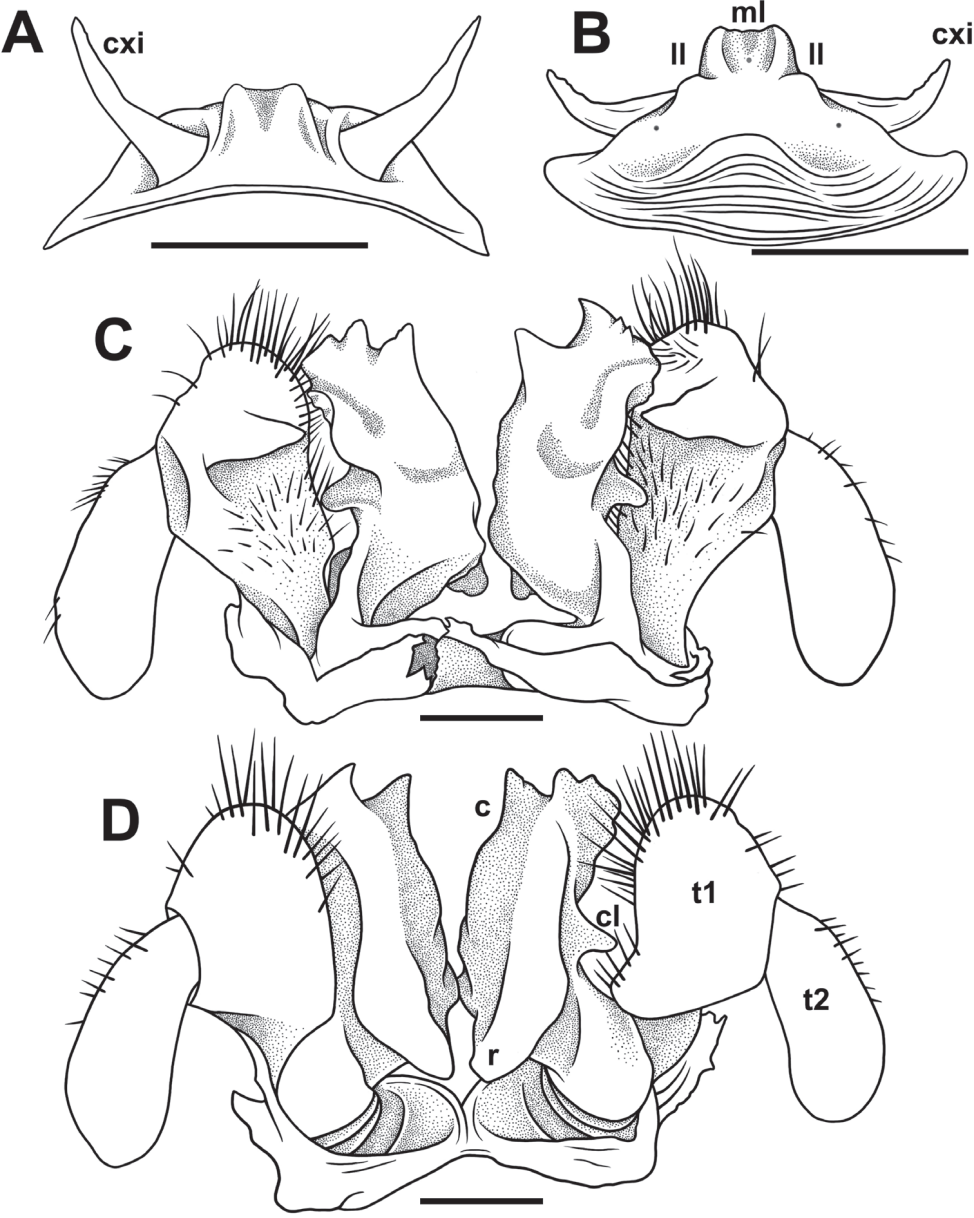
*Nepalellasiamensis* sp. nov., ♂ holotype (CUMZ) **A, B** anterior gonopods, front and caudal views, respectively **C, D** posterior gonopods, caudal and front views, respectively. Abbreviations: **c** colpocoxite, **cl** lateral lobe, **cxi** coxites, **ll** lateral lobules, **ml** median lobe, **r** rounded bulge, **t1** telopoditomere 1, **t2** telopoditomere 2. Scale bars: 0.2 mm.

**Figure 5.**
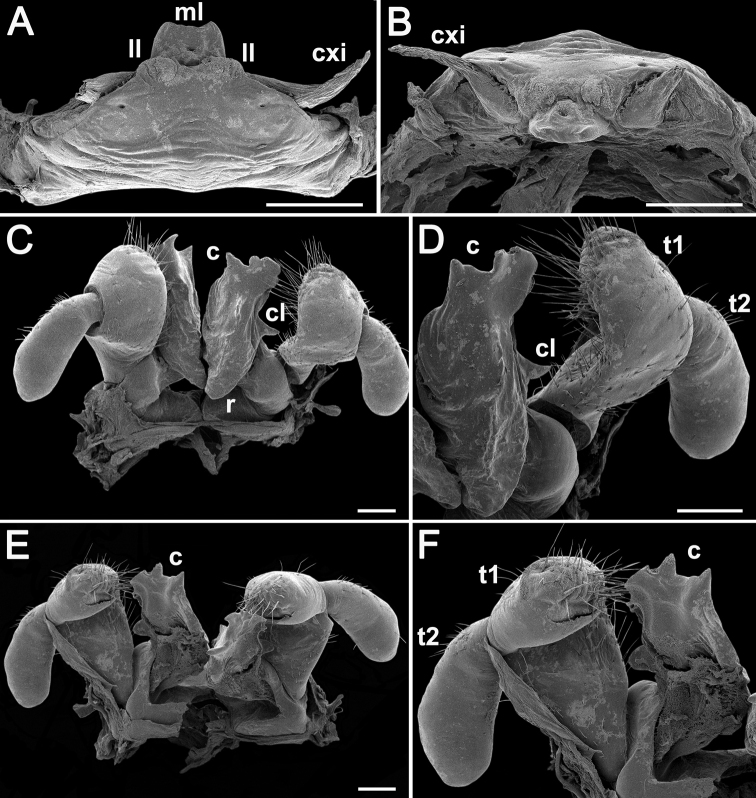
*Nepalellasiamensis* sp. nov., SEM ♂ holotype (CUMZ) **A, B** anterior gonopods, caudal and superior views, respectively **C, E** posterior gonopods, front and caudal views, respectively **D, F** left gonopod, front and caudal views, respectively. Abbreviations: **c** colpocoxite, **cl** lateral lobe, **cxi** coxites, **ll** lateral lobules, **ml** median lobe, **r** rounded bulge, **t1** telopoditomere 1, **t2** telopoditomere 2. Scale bars: 0.2 mm.

Differs from the congeners by ♂ femora 3 and 4 each with a small mushroom-like protuberance (**mp**) ventrally (Fig. 3C); ♂ coxa 10 with a conspicuous horn-shaped process (**h**) dorsally (Fig. 3E, F); ♂ coxa 11 with a small, medial, digitiform process (**m**) and a high, basal, funnel-shaped process (**b**) (Fig. 3G, H); anterior gonopod sternum carrying a median lobe and two small lateral lobules (Figs 4A, B, 5A), coupled with posterior gonopod equipped with a foot-shaped colpocoxite (**c**) and a rounded bulge (**r**) at base in frontal view (Figs 4C, D, 5C, D).

##### Description.

Length of holotype ca 33 mm, maximum width 3.2 mm. Coloration light brown (Fig. 2A, B, D, E, G, H); head light brown, venter and legs light yellowish to pallid (Fig. 2C, F, I). Eye patches and antennae brownish black (Fig. 2A, C).

In width, collum < segment 2 < 3 < head with genae = segment 4 < 5 < 6 < 7 = 20; thereafter, body very gradually tapering towards telson.

Body with 30 segments (29 pleurotergites with free sternites, plus telson, or “rings”, in terms of Enghoff et al. (1993, 2015)).

Head densely setose, clypeolabral region slightly convex. Eye patches triangular, each composed of 27 and 28 convex ommatidia (Fig. 2A, C).

Antennae very long and slender (Figs 2A, 3A), reaching past body segment 6 when stretched posteriorly; antennomere 7 with four apical cones.

Gnathochilarium without promentum (Fig. 3A).

Collum as usual (for heterochordeumatoideans), obcordate in shape, with rudimentary paraterga (Fig. 2A). Tegument smooth, shining, only prozonae distinctly and densely striolate transversely (Fig. 2D, E). Metatergal setation 3 + 3, typical of Chordeumatida; macrochaetae long, rather thick, pointed, placed on clear knobs (Fig. 2A, B, D, E, G, H); stricture between pro- and metazona shallow, inconspicuous (Fig. 2A, D, E, G, H). Paraterga poorly developed, with small dorsolateral bulges in anterior part of body (Fig. 2B), following segments rather regularly rounded in dorsal view (Fig. 2D, E, H).

CIX (ring 15) = 0.62; MIX (ring 15) = 0.87; MA (ring 15) ≈ 145°; PIX impossible to evaluate due to insufficiently developed paraterga. Axial suture distinct, pallid, as usual (Fig. 2B, D, E, H).

♂ legs long and slender, ca 1.5 times as long as midbody height. Legs 1 and 2 slightly reduced, tarsi with usual ventral brushes, but without papillae; ♂ coxa 2 with a distal mediocaudal cone perforated by gonopore orifice. All following legs conspicuously papillate on ventral face of tarsi (Fig. 3C, D, E, G, I). ♂ legs 3–7 distinctly and increasingly crassate, pairs 3 and 4 particularly so. Femora 3 and 4 each with a small, but evident mushroom-shaped protuberance (**mp**) at midway ventrally (Fig. 3C). Coxa 7 with a small, but evident distoventral digitiform outgrowth (Fig. 3D).

♂ legs 10 and 11 each with a small coxal gland (**cg**) (Fig. 3E–H); each coxa 10 dorsally with a large horn-shaped process (**h**) conspicuously enlarged at base (Fig. 3E, F); each coxa 11 with a small, medial, digitiform process (**m**) and a high, basal, funnel-shaped process (**b**) (Fig. 3G, H); prefemur 11 with a small parabasal process (**p**) ventrally (Fig. 3G, H). Claws simple, rather long.

Anterior gonopods (♂ leg-pair 8) very strongly reduced, sternum with a median lobe (**ml**) distally in oral view and with two small lateral lobules (**ll**); coxites (**cxi**) long, slender and horn-shaped (Figs 4A, B, 5A, B).

Posterior gonopods (♂ leg-pair 9) (Figs 4C, D, 5C, D, E, F) hypertrophied, each with a prominent, foot-shaped colpocoxite (**c**), this being higher than telopodite, and with three evident longitudinal lamellae in caudal view; a rather conspicuous lateral lobe (**cl**) at midway in caudal view; with a rounded bulge (**r**) at base in frontal view; telopoditomere 1 (**t1**) particularly strongly setose on posterior face, expanded apically, telopoditomere 2 (**t2**) subpyriform, likewise voluminous, only slightly setose laterally in basal half.

##### Remark.

The specimen was collected by hand while it was moving very fast on the leaf litter surface. The type locality is situated in a dipterocarp forest on the side of a road near the Ta Phraya Waterfall. The species was found syntopically together with *Antheromorphauncinata* (Attems, 1931) (Paradoxosomatidae, Polydesmida) (Likhitrakarn et al. 2016).

### ﻿Key (after adults) to the known species of *Nepalella*, modified after Golovatch et al. (2006b)

**Table:** 

1	Adults with 28 body segments: 27 pleurotergites including telson	***N.phulcokia***
–	Adults with 30 body segments including telson	**2**
2	Body length ≥ 27 mm, width 2.5–3.5 mm	**3**
–	Body length ≤ 26 mm	**10**
3	Midbody paraterga well developed, PIX(15) = 0.17–0.62	**4**
–	Midbody paraterga poorly developed, PIX(15) impossible to evaluate	**5**
4	Body length 27–35 mm, width 3.2–3.5 mm; coloration rather pale; each eye patch with 26 ommatidia; ♂ femora 3 and 4 each with a mushroom-like protuberance ventrally	***N.lobata***
–	Body length 36–38 mm, width 2.6–2.8 mm; coloration light brown; each eye patch with 8–11 ommatidia; ♂ legs 3 and 4 without such modifications	***N.jinfoshan***
5	Each eye patch ≥ 25 ommatidia	**6**
–	Each eye patch with 10–17 ommatidia	**7**
6	Each eye patch with 27–28 ommatidia; coloration light brown; Sa Kaeo Province, Thailand	***N.siamensis* sp. nov.**
–	Each eye patch with 25 ommatidia; coloration dark brown; Yunnan, China	***N.magna***
7	♂ legs 2.0 times as long as midbody height; ♂ coxa 10 with a large process distoventrally; anterior gonopod sternum with a very large and broad median lobe	***N.wangi***
–	♂ legs 1.4–1.8 times as long as midbody height; ♂ coxa 10 without such modifications; anterior gonopod sternum with either a small or an otherwise modified process	**8**
8	Body particularly large, ≥ 40 mm long; antennae very long, reaching past body segment 8 dorsally; anterior gonopod sternum with a high and evident median protuberance and two lateral lobes	***N.grandis***
–	Body smaller, ≤ 40 mm long; antennae shorter, reaching only past body ring 5 dorsally; anterior gonopod sternum with a small median protuberance	**9**
9	♂ legs 1.4 times as long as midbody height; coloration pale brown; ♂ femora 3 and 4 each with a small mushroom-shaped protuberance ventrally	***N.marmorata***
–	♂ legs 1.8 times as long as midbody height; coloration entirely pallid to light yellowish; ♂ legs 3 and 4 without such modifications	***N.grandoides***
10	Body pallid, but eye patches and antennae pigmented; body 2.6–2.7 mm wide due to paraterga well developed, in the form of distinct dorsolateral keels; tergal setae long; ♂ legs 3–7 not enlarged; Myanmar	***N.pallida***
–	Body either entirely pallid (cavernicole) or distinctly pigmented, eye parches and sometimes also antennae pigmented; body width ≤ 2.3 mm, paraterga largely poorly developed, like indistinct dorsolateral swellings; tergal setae medium-sized at most; ♂ legs 3–7 very often crassate	**11**
11	Body length ≥ 18 mm, width ≥ 1.9 mm; coloration uniformly brown, ♂ coxa 10 without distinct processes	**12**
–	Never all these three characters combined	**14**
12	Anterior gonopod sternum with a narrow and acute median process; only ♂ femur 4 roundly gibbose ventrally; Thailand	**13**
–	Anterior gonopod sternum with a round and broad median process; ♂ femora 3 and 4 each with a fungiform protuberance ventrally; Nepal	***N.gunsa***
13	Body length 24 mm, width 2.3 mm; posterior gonopods with colpocoxites divided distally into three branches; ♂ coxa 10 with two large processes distoventrally	***N.taiensis***
–	Body length 17 mm, width 2.0 mm; posterior gonopods with colpocoxites protruded distally and bend down; ♂ coxa 10 with a rather small process distoventrally	***N.inthanonae***
14	Body entirely pallid; ommatidia < 9, reduced, only slightly pigmented and widely separated; cave in Guizhou Prov., China	**15**
–	Body pigmented, > 20 dark and compact ommatidia	**16**
15	Body length 18 mm, width 1.6 mm; each eye patch with nine ommatidia; ♂ legs 3–7 not modified; anterior gonopod sternum with two short, acute, paramedian processes	***N.caeca***
–	Body length 20–26 mm, width 1.5–2.3 mm; each eye patch with 4–6 ommatidia; ♂ legs 3–5 distictly crassate; anterior gonopod sternum without median process	***N.troglodytes***
16	Body 2.2 mm wide, paraterga well developed, shoulder-shaped; ♂ femur 4 with a distal knob subtending a distal depression on ventral side; Yunnan, China	***N.griswoldi***
–	Body width usually ≤ 1.9 mm; paraterga moderately to poorly developed; ♂ femur 4 either unmodified or modified otherwise	**17**
17	Tergal setae short and blunt; ♂ legs 3–7 crassate, but without further modifications; posterior gonopod telopodite relatively strongly reduced, much shorter than colpocoxites; Yunnan	***N.pianma***
–	Tergal setae short to medium-sized, acute; at least some of ♂ legs 3–7 usually with modifications; telopodite of posterior gonopods hypertrophied, (sub)equal in height to colpocoxite	**18**
18	Body width 1.8–2.0 mm; ♂ legs 3–7 with tarsal papillae and dorsally inflated prefemora; Yunnan	***N.kavanaughi***
–	Body width usually ≤ 1.9 mm; ♂ legs 3–7 with neither tarsal papillae nor dorsally enlarged prefemora	**19**
19	Tarsal papillae present on most ♂ legs; ♂ prefemur 11 with a long, digitiform, parabasal process; Vietnam	***N.vietnamica***
–	Tarsal papillae absent from ♂ legs; ♂ prefemur 11 devoid of processes	**20**
20	Claw simple; ♂ coxa 10 with a long process distoventrally; ♂ coxa 11 at most with one small process distoventrally, Nepal	**21**
–	Claw complex, with both a minute accessory claw dorsally and a long setoid filament ventrally at base; ♂ coxae 10 devoid of processes, ♂ coxa 11 with two small processes distoventrally; Myanmar	***N.birmanica***
21	Coloration ochraceous, with four dark, brown, longitudinal stripes	**22**
–	Coloration ochraceous to brownish, with spots, or metazonae dark	**25**
22	Colpocoxites of posterior gonopods divided into three branches or lobes	**23**
–	Colpocoxites of posterior gonopods poorly divided distally into only two short branches	**24**
23	Larger: 16–17 mm long, 1.8–1.9 mm wide; colpocoxite of posterior gonopods divided into three lobes; ♂ coxa 10 with a C-shaped process	***N.tragsindola***
–	Smaller: 10–12 mm long, 1.0–1.3 mm wide; colpocoxite of posterior gonopods divided into two lobes and a slender acuminate branch (solenomere?); ♂ coxa 10 with a coniform process topped by a rounded, microgranulate bulge	***N.gairiensis***
24	Larger: 17 mm long, 1.6 mm wide (♂); both branches of colpocoxite very short and erect; ♂ coxa 10 with a bifid process	***N.ringmoensis***
–	Smaller: 11–14 mm long, 1.3–1.5 mm wide (♂, ♀); middle branch of three unequal branches of colpocoxite directed medially; ♂ coxa 10 with a subtruncate process	***N.deharvengi***
25	♂ coxa 10 with a straight, apically truncate process; ♂ prefemora 3–7 each with a distoventral knob; ♂ coxa 11 without gland, but with a small distomedial process	***N.thodunga***
–	♂ coxa 10 with a curved, apically acuminate process; ♂ prefemora 3–7 either unmodified or only third and fourth with distoventral knobs; ♂ coxa 11 at most with a small gland, devoid of any processes	**26**
26	♂ coxa 10 with a strong unciform process directed caudally; ♂ femora 3–7 each with a ventral fungiform protuberance at midway	***N.taplejunga***
–	♂ coxa 10 with a strong unciform process directed laterad; ♂ femora 3–7 unmodified	**27**
27	Larger: ca 14 mm long, 1.4–1.5 mm wide; tergal setae medium-sized; ♂ prefemora 3 and 4 each with a distoventral knob	***N.khumbua***
–	Smaller: ca 10 mm long, 1.0 mm wide; tergal setae short; ♂ legs 3 and 4 without such modifications	***N.jaljalae***

## ﻿Discussion

At the moment, 28 species of *Nepalella* have been described, mostly (22, ca 79%) from Nepal or China. In Nepal, many species have been encountered at very high elevations of 2200–3800 m a.s.l., although the occurrence in montane habitats (>800 m a.s.l.) is typical of most congeners elsewhere. Allopatry prevails, but sympatry or even syntopy of two congeners has occasionally been recorded as well. As the distributions of all species, both epigean and cave-dwelling, tend to be highly localized, narrow endemism is most characteristic. Cavernicoly seems to be restricted to the karsts of the southern half of China alone, whereas more to the south, even in the abundant karsts of Thailand or Myanmar, all *Nepalella* encounters appear to be only epigean and increasingly sporadic (Table 1). Moreover, there seem to be no troglobionts among the Chordeumatida presently known to occur in Thailand or Myanmar, although at least the cave millipede faunas of Thailand and Indochina are quite well studied (e.g., Golovatch 2015; Likhitrakarn et al. 2015, 2016, 2017, 2018, 2020a, 2020b, 2021). The most common group, likewise both highly diverse and abundant, that clearly dominates the subterranean millipede faunas of Southeast Asia together with southern China is long known to be the family Cambalopsidae (Spirostreptida) (Golovatch 2015; Likhitrakarn et al. 2018, 2020a, b, 2021).

Basically, these characteristics and patterns strongly resemble those of many groups of Diplopoda such as the orders Polydesmida (4 families, 8 genera), Chordeumatida (3 families, 3 genera), Callipodida (3 families, 3 genera), Spirostreptida (2 families, 3 genera), Glomerida (1 family, 1 genus), and Julida (1 family, 1 genus) encountered in caves of southern China (Golovatch and Liu 2020). Thus, it is there that caves appear to be exceptionally rich in millipedes, often with 5–6 diplopod species, mostly very local endemics and presumed troglobionts (Golovatch 2015), occurring per cave. The animals are largely characterized by pronounced troglomorphic features such as reduced and mostly unpigmented eyes, unpigmented bodies, thinner and more delicate teguments, clearly elongated appendages (antennae, legs, claws, tergal outgrowths etc.), and often also the so-called “cave gigantism” (Liu et al. 2017a).

A few *Nepalella* species are among the largest Chordeumatida globally (e.g., *N.grandis*, which is up to 42 mm long) and nearly all appear to be restricted to subtropical rather than purely tropical environments lying between 23.5° and 34°N (Fig. 1). In contrast, lowland, typically tropical occurrences are only very few: *N.vietnamica* from Vietnam, and both *N.taiensis* and *N.inthanonae* from Thailand (Table 1). The new species, *N.siamensis* sp. nov., definitely joins the trio, at the same time representing the most lowland and the southernmost record of a *Nepalella*.

Liu et al. (2017b) recovered the phylogeny of five species of *Nepalella*, based both on morphological and molecular evidence. Barcoding results revealed that interspecific p-distances amounted to 8.5–15.9%, vs 0–6.8% for intraspecific p-distances. The genus was split into two groups associated with such morphological characters as the presence or absence of a median lobe on the sternum of the anterior gonopods. Because of a limited amount of *Nepalella* material used in that pioneering study, future investigations are required to confirm both hypotheses. There is little doubt that further novelties concerning the species diversity and distribution of *Nepalella* are ahead.

## Supplementary Material

XML Treatment for Nepalella

XML Treatment for Nepalella siamensis
